# Latin American Prevalence of Glaucoma: A Systematic Review and Meta-Analysis

**DOI:** 10.3390/vision9020042

**Published:** 2025-05-05

**Authors:** Denisse J. Mora-Paez, Jaime Guedes, Dillan Cunha Amaral, Marcelo Alves Ferreira, Bruno F. Fernandes, Sacha F. Pereira, Bruno Botton, Alisha Desai, Helena Messinger Pakter, Fabio Lavinsky, Adroaldo Alencar Costa Filho

**Affiliations:** 1 Glaucoma Research Center, Wills Eye Hospital, Philadelphia, PA 19107, USA; dpaez@willseye.org (D.J.M.-P.); alisha.desai@students.jefferson.edu (A.D.); ylavinsky@hotmail.com (F.L.); 2 Faculty of Medicine, Federal University of Rio de Janeiro, Rio de Janeiro 21941-971, RJ, Brazil; dillanamaral@ufrj.br (D.C.A.); adroaldoalencar@hotmail.com (A.A.C.F.); 3 Department of Statistics, São Paulo University, São Paulo 05403-000, SP, Brazil; analistaestatistico@gmail.com; 4 Argumento Institute, Montreal, QC J4B 2G6, Canada; bruno.mtl@gmail.com; 5 Faculty of Medical Sciences of Paraiba, FCM, AFYA, João Pessoa 58106-402, PB, Brazil; sachafernandesp@gmail.com; 6 Clínica Schmitt Botton, Santa Maria 97060-360, RS, Brazil; brunobotton@hotmail.com; 7 Sidney Kimmel Medical College, Thomas Jefferson University, Philadelphia, PA 19107, USA; 8 Department of Ophthalmology, Federal University of Rio Grande do Sul, Porto Alegre 90040-060, RS, Brazil; hmpakter@hcpa.edu.br; 9 Department of Ophthalmology, Hospital of Clinics from Porto Alegre, Porto Alegre 90035-903, RS, Brazil

**Keywords:** meta-analysis, systematic review, glaucoma, prevalence, Latin America

## Abstract

We conducted a systematic review and meta-analysis to estimate the prevalence of all types of glaucoma in Latin America (LATAM) and evaluate potential demographic associations. This study followed PRISMA guidelines and was registered in PROSPERO (CRD42024506330). A comprehensive search of PubMed, SciELO, and Web of Science was conducted to identify population-based or cross-sectional studies reporting glaucoma prevalence in LATAM. Studies were categorized into two groups: Group 1 included general population studies without selection based on visual acuity (VA), and Group 2 included studies limited to individuals with VA < 20/60. Data from five studies in Group 1 (25,288 individuals) and eight studies in Group 2 (29,882 individuals) were analyzed using R software. The pooled prevalence of glaucoma was 4% (95% CI: 1–3%) in Group 1 and 1% (95% CI: 0–1%) in Group 2. No statistically significant associations were found between glaucoma prevalence and sex (*p* = 0.08) or age (*p* = 0.5669). Although our findings highlight the relevance of glaucoma as a public health concern in LATAM, the limited number of available studies and methodological variability reduce the certainty of the estimates.

## 1. Introduction

Blindness and visual impairment are critical public health issues that affect approximately 596 million people worldwide [1]. As the leading cause of irreversible blindness and the second leading cause of blindness, glaucoma imposes a significant societal burden worldwide [2,3,4]. Glaucoma comprises a diverse group of optic neuropathies characterized by the gradual degeneration of retinal ganglion cells and their axons. This degeneration results in varying degrees of irreversible visual field loss over time [1,5]. In 2020, the number of people with glaucoma worldwide was estimated to be more than 70 million, with a predicted increase to more than 110 million by 2040 [6]. The population-based prevalence of glaucoma varies widely across individual studies due to various risk factors present in samples studied, including age, sex, and geographic location [6,7,8,9].

Historical epidemiological studies show significantly different results from recent studies due to updated study designs and diagnostic methods [10,11]. According to United Nations data, Latin America (LATAM) accounts for 8.3% of the world’s population [12]. Although the prevalence of glaucoma has been estimated globally by meta-analyses of epidemiological population-based studies [6], there are few population-based studies on glaucoma prevalence in the LATAM region. Instead, the estimated prevalence of open-angle glaucoma (OAG) has been calculated using studies conducted on Hispanic populations in the United States [13,14,15]. Since 2000, there have been additional studies on the prevalence of glaucoma in individual LATAM countries [15,16,17]. However, to our knowledge, no meta-analysis covering the prevalence of glaucoma in the LATAM region has been published. Based on the need to establish the impact of glaucoma in LATAM, we carried out this systematic review and meta-analysis to assess the prevalence of glaucoma in LATAM populations.

## 2. Materials and Methods

This meta-analysis was performed according to the guidelines of the Declaration Preferred Reporting Items for Systematic Reviews and Meta-Analysis (PRISMA) and the recommendations of the Cochrane Collaboration [18,19]. The protocol was registered in the International Prospective Register of Systematic Reviews (PROSPERO) under registration number CRD42024506330.

### 2.1. Elibility Criteria

Studies that met the following eligibility criteria were included: (1) cohort or cross-sectional studies; (2) population-based studies of any type of glaucoma conducted in LATAM countries; (3) studies with clear definitions of random or clustered sampling procedures; (4) studies including people aged ≥ 18 years; and (5) studies from which the prevalence data for glaucoma in an LATAM population could be extracted or calculated. Exclusion criteria were as follows: (1) studies that were not general population-based (e.g., hospital-based or clinical-based); (2) studies that did not include the number of subjects with glaucoma; (3) studies that used data from the authors’ previous publications.

### 2.2. Information Source

Two authors (M.F. and B.F.) independently searched PubMed, Web of Science, and SciELO from inception to January 2024. The references from all included studies were also searched manually for any additional studies that could meet the eligibility criteria. Eventual conflicts were resolved by consensus among the authors.

### 2.3. Search Strategy

The following terms were used in our search strategy: “prevalence” and “glaucoma”. Alternative terms for “prevalence” and “survey” were also used. When searching Web of Science, a country/region filter was used, limiting the results to the scope of LATAM. Additional steps and filter strategies used in PubMed and SciELO searches are detailed in Supplementary File S1. There were no publication date or language restrictions in our electronic search.

### 2.4. Study Selection

We imported search results into Zotero software. Duplicate entries were excluded. Two independent authors (D.A. and J.G.) applied the eligibility criteria to screen titles and abstracts. After that, full texts of potentially eligible studies were retrieved. 

### 2.5. Data Extraction

Two authors (D.A. and J.G.) extracted the following data from selected studies: (1) first author; (2) year of study; (3) mean age of the sample (in years); (4) total sample size; (5) total prevalence of glaucoma; and (6) male and female prevalence. Also, the same authors (D.A. and J.G.) collected pre-specified baseline characteristics and outcome data, and recorded these in an Excel template.

### 2.6. Endpoints and Subgroup Analysis

For the prevalence analysis, we divided the studies into two groups. The first group (Group 1) included studies that applied no visual acuity (VA)-based restrictions in their eligibility criteria [15,16,17,20,21], while the second group (Group 2) comprised studies that selected participants exclusively based on having a VA of less than 20/60 [22,23,24,25,26,27,28]. This division was not only based on our methodological considerations but also reflected the categorization explicitly or implicitly adopted by the original studies themselves. We separated the studies in this manner because Group 2 consisted primarily of blindness surveys targeting individuals with visual impairment, which are inherently not representative of the general population. Analyzing these studies separately allowed us to avoid underestimating the overall glaucoma prevalence that could occur if fundamentally different sampling methods were pooled together.

The outcomes of interest include (1) the overall prevalence of glaucoma in the LATAM population, and in male and female subgroups using the studies of Group 1; and (2) the prevalence of glaucoma among individuals with moderate vision impairment (MVI) (VA < 20/60 and ≥20/200), severe vision impairment (SVI) (VA < 20/200 and ≥20/400), and blindness (VA < 20/400) using the studies of Group 2.

### 2.7. Statistical Analysis and Risk of Bias Assessment

We selected the prevalence of glaucoma in the LATAM population as the primary outcome. All statistical analyses were done using R software, version 4.3.1. The “meta” package was utilized to generate forest plots illustrating the prevalence of glaucoma in LATAM across individual studies, including their respective weights and the pooled prevalence with associated 95% confidence intervals (CI). Heterogeneity between studies was quantified using I^2^ statistic [29]. I^2^ values > 50% were considered to indicate significance for heterogeneity [30]. A funnel plot was used to assess potential bias and minor/significant study effects [31]. Asymmetry was evaluated using Begg’s test [32]. A meta-regression model was employed to examine the potential variation in glaucoma prevalence with age using the mean age reported in included studies [33]. However, this analysis was limited by the availability of mean age data in only six studies. The quality of each selected article was assessed using the checklist developed by Downs and Black, and each included article was assessed and scored on a 10-item scale [34]. The prevalence of glaucoma in LATAM was estimated using the “random effects” calculation method. The original proportions were transformed into “logit” values (log(p/(1 − p))), and the weights were calculated based on the inverse of the variance of proportions.

## 3. Results

### 3.1. Study Selection and Baseline Characteristics

**Figure 1:**
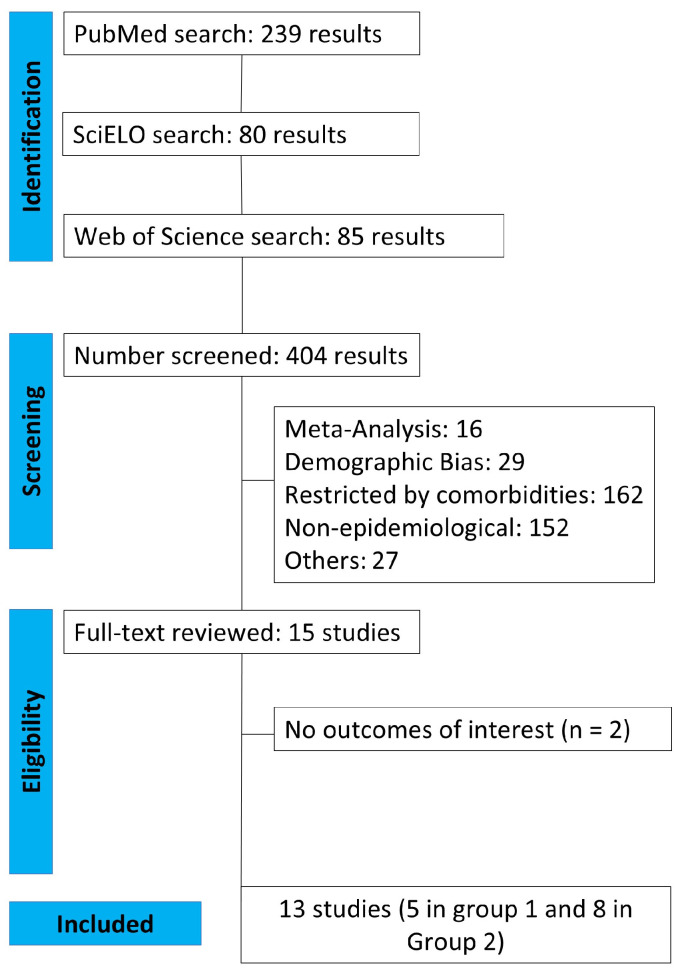
PRISMA flow diagram of the study screening and selection process.

An initial search identified 404 potential articles, and 13 studies remained after removing duplicate records and studies that did not meet the eligibility criteria (Figure 1).

Among these 13 studies, five were in Group 1 [15,16,17,20,21] with a total of 25,288 individuals, and eight in Group 2 [22,23,24,25,26,27,28] with 29,882 individuals. The subject count ranged from 1261 (Mitchell, 2008 [21]) to 18,892 (Castellanos-Perrilla, 2015 [16]); combined, a total of 55,170 subjects were analyzed [16,21]. The characteristics and quality assessment scores (maximum of 10) of the included studies are indicated in Table 1.

### 3.2. Pooled Analysis

#### 3.2.1. Prevalence of Glaucoma in the LATAM Population

**Figure 2:**
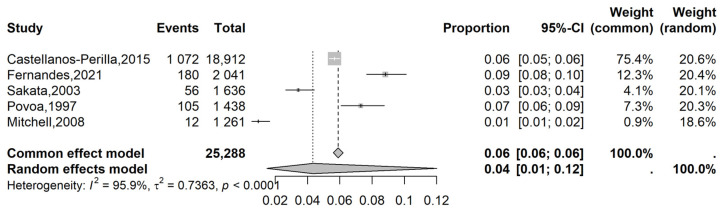
Forest plot of eligible studies on the overall Latin American prevalence of glaucoma in the general population [15,16,17,20,21].

**Figure 3:**
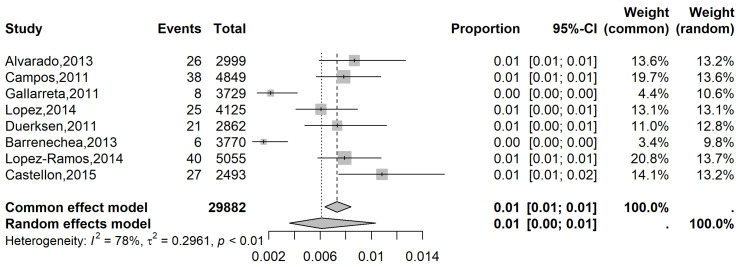
Forest plot of eligible studies on the overall Latin American prevalence of glaucoma in patients with visual acuity < 20/60 [22,23,24,25,26,27,28].

The analysis of all glaucoma cases in Group 1 showed a prevalence of 4% (95% CI: 1 to 12%; I^2^: 96%; Figure 2) while in Group 2, the prevalence was 1% (95% CI: 0 to 1%; I^2^: 78%; Figure 3). The meta-regression model did not reveal a significant association between age and prevalence (*p* = 0.56).

#### 3.2.2. Subgroup Analysis

**Figure 4:**
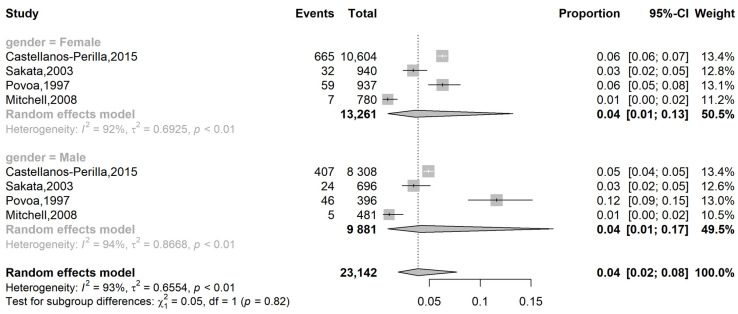
Forest plot of eligible studies on the differences in the prevalence of glaucoma in Latin America based on sex [15,16,20,21].

We observed methodological heterogeneity between studies by subgroup, where I^2^ = 92% and I^2^ = 94% for female and male subgroups, respectively. The analysis of the prevalence of glaucoma in LATAM based on sex was reported in all studies of Group 1. The pooled analysis showed a prevalence of 4% (95 CI: 1 to 13%) in females and 4% (95 CI: 2 to 8%) in males with a random-effect model (Figure 4). There was no statistically significant difference based on sex (*p* = 0.8).

**Figure 5:**
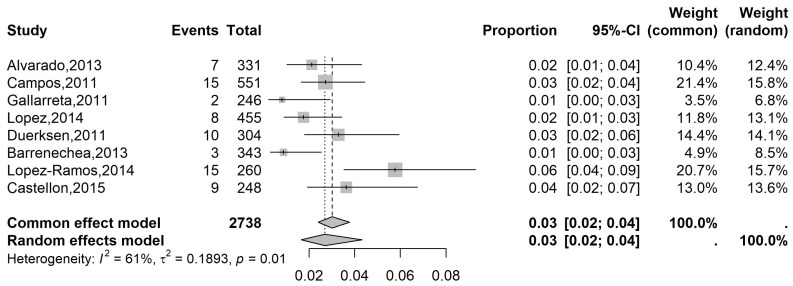
Forest plot of eligible studies on the Latin American prevalence of glaucoma in individuals with moderate vision impairment [22,23,24,25,26,27,28].

**Figure 6:**
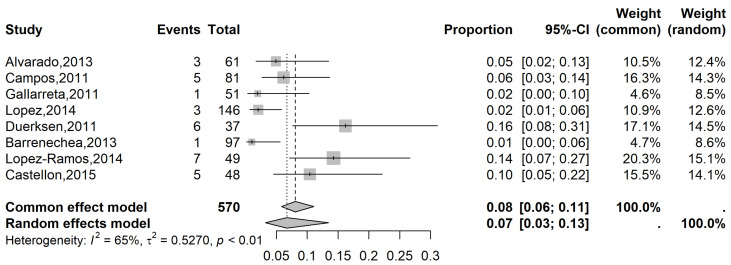
Forest plot of eligible studies on the Latin American prevalence of glaucoma in individuals with severe vision impairment [22,23,24,25,26,27,28].

**Figure 7:**
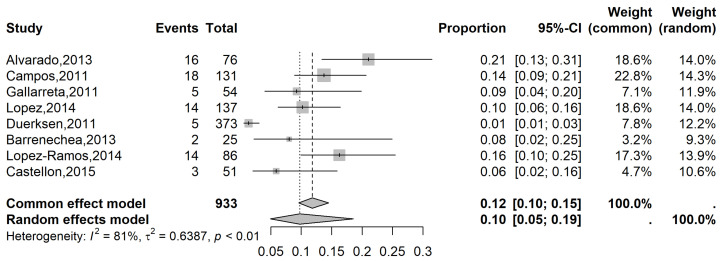
Forest plot of eligible studies on the Latin American prevalence of glaucoma in blind individuals [22,23,24,25,26,27,28].

The pooled prevalence of glaucoma in the LATAM population with MVI was 3% (95% CI: 2 to 4%), while the pooled prevalence was 7% (95% CI: 3 to 13%) in the population with SVI (Figure 5 and Figure 6, respectively) and 10% (95% CI, 5 to 19%) in the blindness population (Figure 7).

### 3.3. Quality and Evidence Assessment and Risk of Bias

**Figure 8:**
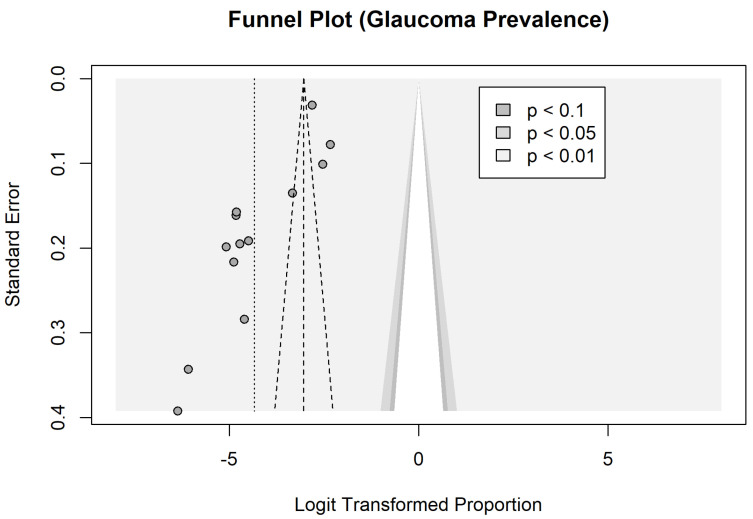
Funnel plot analysis for quality and evidence assessment and risk of bias in 13 studies on the prevalence of glaucoma in Latin America.

All the studies included in our analysis were cross-sectional or population based. Of these, three studies scored a 10 in quality assessment, five studies scored a 9, and five studies received a score of 8. The funnel plot and Begg’s test for asymmetry showed homogeneity (z = −1.59; *p* = 0.11), indicating that any potentially biased outliers did not significantly affect estimates (Figure 8).

## 4. Discussion

Based on this meta-analysis of 13 population-based studies, which comprised a total of 55,170 subjects, the overall prevalence of glaucoma in the LATAM population in Group 1 was 4% (95% CI: 1 to 12%), and in Group 2 was 1% (95% CI: 0.00 to 0.01%).

This estimation of 4% in Group 1 is similar to the previous report of 3.4% LATAM and Caribbean primary open-angle glaucoma (POAG) prevalence in 2021 and previous systematic review and meta-analysis results of 3.90% LATAM and Caribbean prevalence of POAG in 2021 [6,35]. Both reviews were based on studies that did not use impairment evaluation for selecting participants. As a result, the subgroup analyses, which similarly lacked impairment evaluation, yielded comparable results. However, in Group 2, the reported prevalence of low vision was 1%. This discrepancy suggests that focusing solely on patients with VA < 20/60 may underestimate the overall prevalence of glaucoma.

Previous reports suggest that the role of sex as a potential risk factor for glaucoma remains controversial [7,36,37,38,39,40,41,42,43]. Previous studies have identified male sex as a potential risk factor for glaucoma, citing males’ longer axial length and deeper anterior chamber depth as contributing factors [44,45,46]. However, additional studies have identified female sex as a potential risk factor for glaucoma [40,43,47], suggesting that anatomical predispositions and fluctuations in female sex hormones could influence IOP and vascular resistance, thereby potentially impacting optic nerve head circulation [37]. In our study, the difference between sexes was not a significant risk factor for glaucoma (*p* = 0.8). Thus, the impact of sex on glaucoma is still inconclusive [11,37]. With the global population aging, and the prevalence of glaucoma and glaucoma-related blindness on the rise, there is a growing need to conduct further, more extensive studies on this topic with the overall goal of raising awareness and enhancing our understanding of sex disparities in glaucoma health. Such efforts will also aid in identifying at-risk populations and optimizing resource allocation in order to improve care for patients with glaucoma.

To estimate the prevalence of blindness and visual impairment in Group 2, eight studies were examined. The prevalence of blindness found in Uruguay is the lowest for all Rapid Assessment of Avoidable Blindness (RAAB) studies performed in Latin America thus far [24]. The significant occurrence of blindness caused by glaucoma underscores the need for early detection to halt disease progression. The analysis of the prevalence of MVI, SVI, and blindness resulted in the assessment of 2738, 570, and 933 subjects, respectively. Thus, in Group 2, the pooled prevalence of MVI due to glaucoma was 3% (95% CI: 2 to 4%), while SVI was 7% (95% CI: 3 to 13), and blindness was 10% (95% CI, 5 to 19%). This analysis shows that the prevalence of glaucoma increases as visual impairment worsens. Patients with MVI due to glaucoma may be unaware of the condition due to subtle or asymptomatic symptoms. As vision deteriorates, the rate of SVI and blindness due to glaucoma increases, possibly due to disease progression without adequate treatment or resistance to interventions, resulting in total vision loss [48,49].

Although the included studies did not report on specific public health actions or preventive strategies, our findings highlight the need for such measures to be prioritized in LATAM. Given the asymptomatic progression of glaucoma and its high prevalence, we suggest that future regional efforts include widespread screening programs, public awareness campaigns, and improved access to diagnostic and treatment services. Implementing such initiatives could facilitate earlier detection and management, ultimately reducing the burden of glaucoma-related visual impairment in the region. These results are especially relevant for ophthalmologists, public health officials, policymakers, and researchers aiming to understand the regional burden of glaucoma. However, the findings also reveal critical gaps in data standardization, geographic coverage, and reporting of demographic subgroups. Future studies should focus on longitudinal trends, evaluate regional screening and treatment programs, and incorporate detailed demographic and clinical data to support more targeted and effective glaucoma control strategies across LATAM.

Although the results of this study provide significant insights into the prevalence of glaucoma in LATAM, it is important to consider its limitations. The included studies do not encompass all countries in LATAM, as shown in Table 1, and they are limited in representing the diverse population characteristics of their countries of origin. For example, the study by Sakata et al. focuses on a South Brazilian population, which, in a vast and diverse country like Brazil, may not fully capture the regional differences present across the country [15]. There was a notable degree of heterogeneity across studies, highlighting substantial inconsistency in the definitions of glaucoma (Table 2) and study design. Recent studies have increasingly sought to standardize these definitions to improve comparability and support more reliable prevalence estimates across populations [50,51,52]. Although the overall heterogeneity in this meta-analysis was high (I^2^ = 99%), it is common for meta-analyses of prevalence studies to have very high heterogeneities, with I^2^ values usually over 90% [53,54,55]. The random effects models employed in our analysis aimed to address and mitigate this limitation. Random effects models are preferred over fixed-effect models in medical research because they accommodate varying true effects and address unexplained heterogeneity [56,57,58]. Subgroup analyses by sex were underpowered and did not distinguish between glaucoma subtypes (e.g., POAG vs. primary angle-closure glaucoma [PACG]), limiting the ability to draw meaningful conclusions. This limitation is further compounded by the fact that many included studies lacked detailed subgroup data on variables such as age, race, urban versus rural residence, and specific glaucoma subtypes like PACG, which has been shown in previous reports to have a lower prevalence compared to POAG [6,58]. Moreover, due to LATAM being an ethnically diverse region, the inclusion of various ethnic groups (e.g., Black, White, and Asian) may mask potential differences in glaucoma prevalence among these groups. To address these limitations, we conducted a quality assessment, applied a random effects model and meta-regression, and performed funnel and subgroup analyses.

Future research should address these gaps through standardized, population-based studies that integrate clinical, demographic, and policy-level data to improve glaucoma surveillance and guide evidence-based healthcare planning in the region.

## 5. Conclusions

In summary, this systematic review and meta-analysis, which included five studies in Group 1 and eight studies in Group 2 encompassing a total of 57,170 subjects, found that the prevalence of glaucoma in LATAM is 4% in Group 1 and 1% in Group 2. As glaucoma remains a worldwide vision-threatening disease with high prevalence, future research is needed to continue to investigate the impact of different variables, such as glaucoma definition, glaucoma type, and sex, on glaucoma prevalence in LATAM. Understanding the differences in glaucoma prevalence between geographical regions worldwide is not only vital for the establishment of public health policies, but will also provide greater insight into the causes behind glaucoma health disparities.

## Figures and Tables

**Table 1:** Baseline Characteristics of included studies.

Author	Year	Location	Age Inclusion	Mean Age	N Total	N Total (M/F)	N Glaucoma	N Glaucoma (M/F)	Quality Score
Group 1									
Sakata	2003	Brazil	40 at least	53.7	1636	696/940	56	24/32	10
Povoa	1997	Brazil	40 at least	58.2	1438	396/937	105	46/59	10
Mitchell	2008	Venezuela	NA	NA	1261	481/780	12	5/7	8
Castellanos-Perilla	2015	Colombia	60 at least	69.11	18,912	8308/10,604	1072	407/665	10
Fernandes	2021	Brazil	45 at least	60.33	2041	NA	180	NA	8
Group 2									
Gallarreta	2011	Uruguay	50 at least	NA	3729	1571/2158	8	NA	9
Lopez	2014	Panamá	50 at least	NA	4125	NA	25	NA	8
Alvarado	2013	Honduras	50 at least	NA	2999	NA	26	NA	8
Campos	2011	Peru	50 at least	65.07	4849	2014/2835	38	NA	9
Duerksen	2011	Paraguay	50 at least	NA	2862	NA	21	NA	8
Barrenechea	2013	Argentina	50 at least	NA	3770	1691/2079	6	NA	9
Lopez-Ramos	2014	Mexico	50 at least	NA	5055	NA	40	NA	8
Castellon	2015	Costa Rica	50 at least	64.64	2493	923/1570	27	NA	9

M: male; F: female; NA: not applicable; N: number of patients.

**Table 2:** Presents the definitions of glaucoma as outlined in each study.

Author	Year	Definition of Glaucoma
Group 1		
Sakata	2003	Diagnosis required either: (1) optic disc abnormalities (VCDR ≥ 97.5th percentile in a hypernormal population) plus compatible VF defect; or (2) if VF could not be performed, a severely damaged optic disc (VCDR ≥ 99.5th percentile) alone was sufficient.
Povoa	1997	Referred to second phase if IOP ≥ 21 mmHg and/or at least one of the following: C/D ratio ≥ 0.5, disc asymmetry ≥ 0.2, localized rim thinning, papillary hemorrhage, or pallor. Diagnosis based on comprehensive ophthalmic assessment.
Mitchell	2008	Presumed glaucoma was diagnosed with IOP > 25 mmHg, disc abnormalities (as defined), and nerve fiber bundle loss.
Castellanos-Perilla	2015	Glaucoma status based on self-reported history of diagnosis.
Fernandes	2021	Diagnosis based on anterior segment biomicroscopy, lens status, IOP measurement, and dilated fundus examination.
Group 2		
Gallarreta	2011	NA
Lopez	2014	NA
Alvarado	2013	NA
Campos	2011	NA
Duerksen	2011	Based on afferent pupil defect and corneal edema, VCDR > 0.8, or stone-hard eye on palpation.
Barrenechea	2013	NA
Lopez-Ramos	2014	NA
Castellon	2015	NA

NA: Not Available; IOP: Intraocular Pressure; VF: Visual Field; VCDR: Vertical Cup-to-Disc Ratio; C/D: Cup-to-Disc.

## Data Availability

The original contributions presented in this study are included in the article and Supplementary Material. Further inquiries can be directed to the corresponding author.

## Supplementary Materials

The following supporting information can be downloaded at https://www.mdpi.com/article/10.3390/vision9020042/s1: Supplemental File S1: Full-search database.

## Abbreviations

The following abbreviations are used in this manuscript:

OAG	Open Angle Glaucoma
LATAM	Latin America
PRISMA	Preferred Reporting Items for Systematic Reviews and Meta-Analysis
CI	Confidence Intervals
