# Supplementary material for: Latin American Prevalence of Glaucoma: A Systematic Review and Meta-Analysis
Source: Vision (Basel). 2025 May 5;9(2):42. doi: 10.3390/vision9020042 (PMC12101209; doi:10.3390/vision9020042)
Supplement: Supplementary file 1 [file vision-09-00042-s001.zip › vision-3573569-supplementary.pdf]

Supplementary Materials: Latin American Prevalence of Glaucoma: A Systematic Review and Meta-Analysis

The search for "glaucoma" and "prevalence" on PUBMED yields over 8,000 results. Therefore, the actual search performed included the names of all Latin American countries within the title and abstract of the articles:

PUBMED and Scielo Search Strategy:

(glaucoma AND prevalence) AND (Argentina[Title/Abstract] OR Uruguay[Title/Abstract] OR Brazil[Title/Abstract] OR Chile[Title/Abstract] OR Peru[Title/Abstract] OR Bolivia[Title/Abstract] OR Colombia[Title/Abstract] OR Venezuela[Title/Abstract] OR Ecuador[Title/Abstract] OR Guatemala[Title/Abstract] OR El Salvador[Title/Abstract] OR Nicaragua[Title/Abstract] OR Mexico[Title/Abstract] OR Cuba[Title/Abstract] OR Dominican Republic[Title/Abstract] OR Paraguay[Title/Abstract] OR Haiti[Title/Abstract] OR Costa Rica[Title/Abstract]) Filters: Humans Sort by: Publication Date

An identical strategy was applied to the search in the Scielo database.

In both cases, the resulting sets of articles were reviewed by their titles to exclude non-epidemiological studies or those with hospital-based populations or populations defined by comorbidities. Subsequently, the articles on the resulting list were read to assess their usability.

Web of Science Search Strategy

For the Web of Science database, a comprehensive search was performed to identify studies reporting the prevalence of glaucoma in Latin America. The initial search strategy used the terms **"prevalence" AND "glaucoma"**, and alternative keywords such as **"encuesta"** and **"survey"** were tested to increase sensitivity. This search yielded **2,256 articles**, which were then filtered by country and region to focus on studies conducted in **Latin American countries**, reducing the set to **85**

potentially eligible articles. The search included studies published between **1997 and 2021**, with no restrictions on language.
